# From Leukemia to Solid Tumors, Successful Multidisciplinary Management of Three Distinct Primary Cancers in a Single Patient: A Case Report

**DOI:** 10.1002/ccr3.72800

**Published:** 2026-05-25

**Authors:** Abolfazl Khalafi‐Nezhad, Nazanin Alemzadeh, Mohammad Hossein Anbardar, Zahra Ghanbarinasab

**Affiliations:** ^1^ Department of Hematology, Medical Oncology and Stem Cell Transplantation, Hematology Research Center Shiraz University of Medical Sciences Shiraz Iran; ^2^ Department of Pathology Shiraz University of Medical Science Shiraz Iran

**Keywords:** metachronous cancers, multidisciplinary cancer management, multiple primary malignancies, triple primary malignancy

## Abstract

A 41‐year‐old man developed three distinct primary malignancies—acute myeloid leukemia, male breast cancer, and metastatic colorectal cancer—within 7 years. This rare case highlights the evolving challenge of multiple primary cancers and demonstrates how coordinated multidisciplinary care can preserve curative intent despite successive life‐threatening diagnoses.

## Key Clinical Message

Multiple primary malignancies pose major therapeutic challenges, particularly after intensive prior treatments. Nevertheless, individualized management guided by molecular profiling and multidisciplinary decision‐making, with a consistent curative intent, can achieve successful long‐term outcomes even in patients with sequential advanced malignancies.

**Abbreviations**
AML: acute myeloid leukemia
CT: computed tomography
EGFR: epidermal growth factor receptor
FLAG regimen: fludarabine, high‐dose cytarabine, and granulocyte colony‐stimulating factor
FOLFOX regimen: 5‐fluorouracil, leucovorin, and oxaliplatin
TC regimen: docetaxel and cyclophosphamide

## 1 Introduction

In the evolving landscape of modern oncology, the rising prevalence of multiple primary malignancies, specifically metachronous cases, has emerged as a significant clinical challenge. This trend reflects advances in diagnostic precision, improved surveillance, and the development of novel therapeutic modalities—including chemotherapy, radiotherapy, and targeted agents. Although these advances have meaningfully extended patient survival, prolonged survivorship has also increased the risk of subsequent primary neoplasms [1].

The concept of multiple primary malignancies was first described by Warren and Gates in 1932, marking a significant milestone in the understanding of cancer biology. According to their established criteria, these malignancies are characterized by the occurrence of two or more histologically distinct tumors, each confirmed as malignant and arising independently, rather than representing metastatic spread from a single primary source. Classification is based on the interval between diagnoses: synchronous malignancies are identified within 6 months of the initial diagnosis, whereas metachronous malignancies are detected after a period exceeding 6 months [2].

Although the reported incidence of multiple primary cancers differs across populations, an overall increase has been consistently observed. Their development is thought to arise from a complex interplay of host‐ and tumor‐related factors, including genomic instability, epigenetic changes, immune dysregulation, hormonal imbalance, and alterations in the tumor microenvironment. In addition, disruption of key signaling pathways, such as epidermal growth factor receptor (EGFR), as well as the presence of circulating tumor DNA, has been implicated in their pathogenesis [3].

Beyond these biological mechanisms, a range of environmental and lifestyle‐related factors also contribute to the risk of developing multiple malignancies. Inherited genetic susceptibility, advanced age, obesity, and tobacco use have all been recognized as important contributors [4].

The potential role of cancer treatment in the development of secondary tumors remains an area of ongoing debate. Although chemotherapy, radiotherapy, and hormonal therapy may increase this risk, treatment‐related effects alone are unlikely to fully account for the occurrence of multiple primary cancers [5].

The prognosis of patients with multiple primary malignancies remains a subject of ongoing debate. Several studies have reported that survival outcomes may be comparable to or even better than those observed in patients with a single malignancy. Notably, a longer interval between cancer diagnoses has been associated with improved outcomes, possibly reflecting the benefits of closer clinical surveillance, heightened patient awareness, and earlier detection [2].

In this report, we describe a rare case of a patient diagnosed with three distinct primary malignancies, highlighting the clinical complexity and the importance of long‐term surveillance in cancer survivors.

## 2 Case Presentation

### 2.1 Case History (Part 1)

In 2016, a 41‐year‐old Iranian man was referred to our center with marked leukocytosis (white blood cell count: 103.7 × 10^9^/L), anemia (hemoglobin: 9.7 g/dL), and a platelet count of 150 × 10^9^/L. He was admitted to Namazi Hospital (Shiraz, Iran) for further diagnostic evaluation.

### 2.2 Investigations and Treatment (Part 1)

**FIGURE 1:**
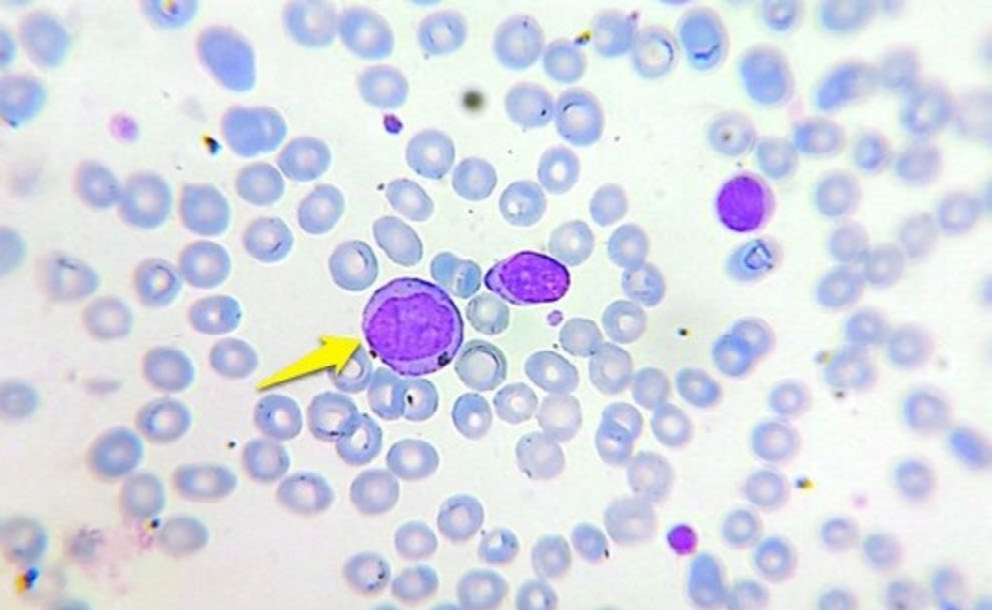
Peripheral blood smear demonstrating circulating blasts (arrows), characterized by a high nuclear‐to‐cytoplasmic ratio and prominent nucleoli.

**FIGURE 2:**
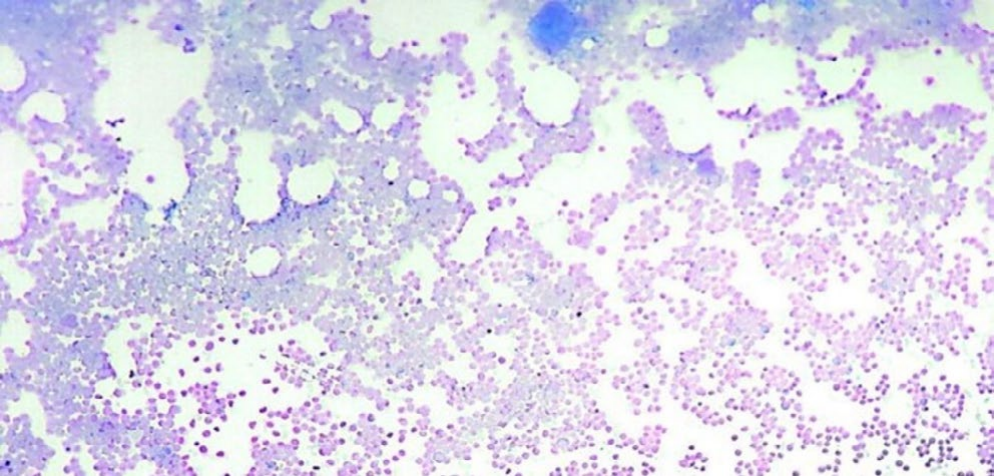
Bone marrow aspiration biopsy at low magnification (×10) showing hypercellular marrow with diffuse infiltration by leukemic blasts.

**FIGURE 3:**
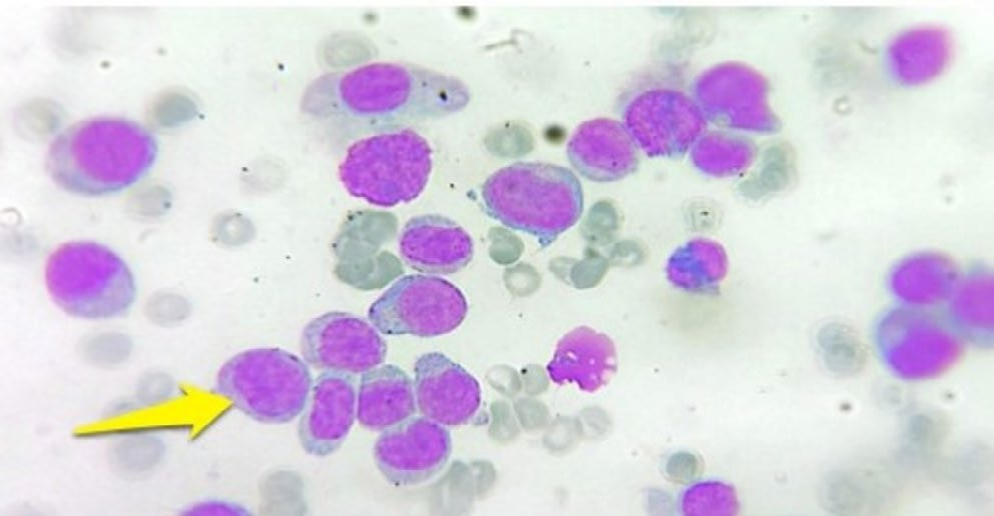
Bone marrow aspiration biopsy at higher magnification (×100) highlighting sheets of myeloblasts with fine chromatin and conspicuous nucleoli (arrows).

A peripheral blood smear showed circulating blasts with a high nuclear‐to‐cytoplasmic ratio and prominent nucleoli (Figure 1). Bone marrow aspiration and biopsy revealed a hypercellular marrow diffusely infiltrated by leukemic blasts, consistent with acute myeloid leukemia without maturation (AML‐M2) (Figures 2 and 3). Flow cytometric analysis confirmed the presence of approximately 20% myeloblasts.

Induction chemotherapy was initiated using the standard 7 + 3 regimen of cytarabine and daunorubicin. However, complete remission was not achieved after two cycles of induction therapy, and the disease was therefore classified as primary refractory AML. The patient subsequently received salvage treatment with fludarabine, high‐dose cytarabine, and granulocyte colony‐stimulating factor (FLAG regimen).

### 2.3 Conclusion and Results (Part 1)

Follow‐up bone marrow evaluation showed a normocellular marrow with fewer than 5% blasts, and flow cytometry demonstrated approximately 1% CD34+/CD117+ immature myeloid cells, findings consistent with remission. Given his high‐risk disease status, the patient subsequently underwent allogeneic hematopoietic stem cell transplantation, which was associated with a favorable clinical outcome.

### 2.4 Case History (Part 2)

Two years later, in 2018, during routine follow‐up, the patient reported a palpable mass in the left breast. He was subsequently evaluated to establish a definitive diagnosis and to assess the possibility of malignancy.

### 2.5 Investigations and Treatment (Part 2)

**FIGURE 4:**
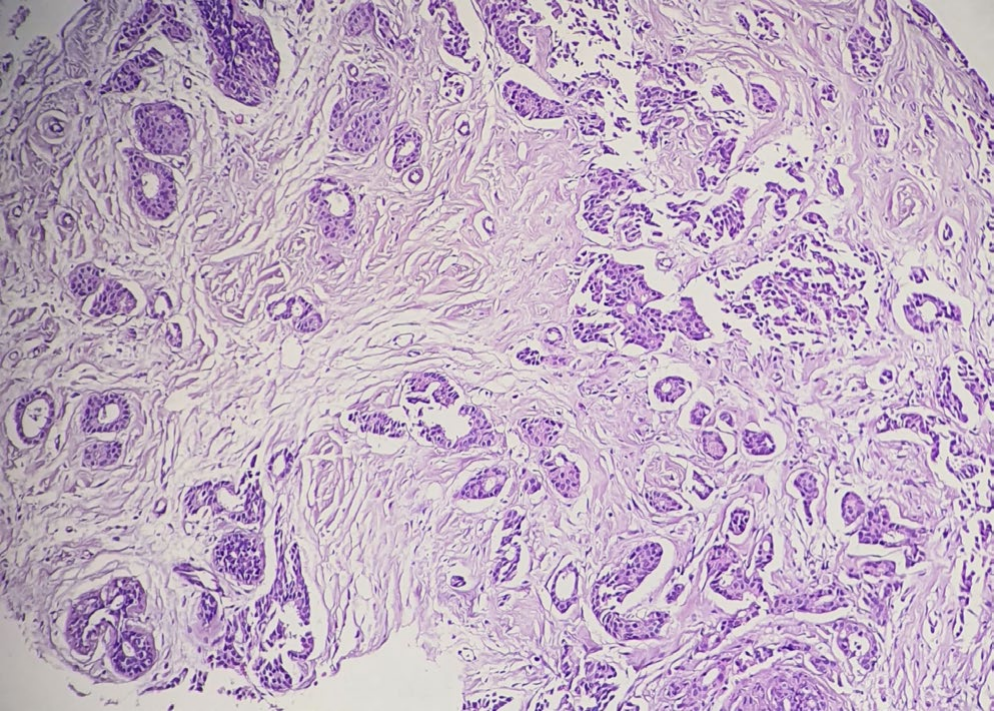
Microscopic section from breast mass shows invasive ductal carcinoma with infiltrating tubular and ductal glands and desmoplasia.

Histopathological evaluation of a biopsy revealed invasive ductal carcinoma, grade II/III, composed of infiltrating ductal and tubular structures with associated desmoplasia (Figure 4). He underwent a left simple mastectomy with sentinel lymph node biopsy. Pathological examination demonstrated involvement of the sentinel lymph node, while surgical margins were free of tumor. Immunohistochemical analysis showed absence of estrogen receptor, progesterone receptor, and HER2 expression. Given the patient's prior exposure to anthracyclines and the associated risk of cardiotoxicity, adjuvant chemotherapy with docetaxel and cyclophosphamide (TC regimen) was initiated, leading to remission.

### 2.6 Case History (Part 3)

In 2023, during ongoing long‐term follow‐up, the patient was found to have iron‐deficiency microcytic anemia. A positive fecal immunochemical test prompted colonoscopic evaluation, which revealed a 6‐cm ulcerated mass in the sigmoid colon.

### 2.7 Investigations and Treatment (Part 3)

**FIGURE 5:**
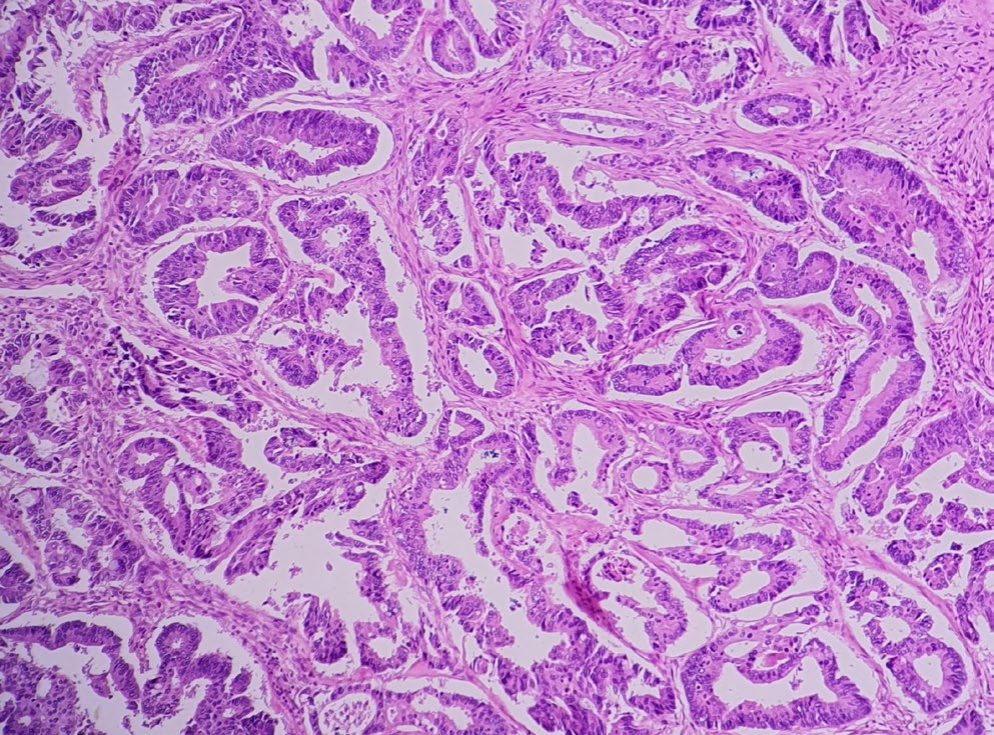
Microscopic section from colon mass shows adenocarcinoma with infiltrative glands and high‐grade dysplasia.

Histopathological examination confirmed a poorly differentiated adenocarcinoma with infiltrative glandular architecture and high‐grade dysplasia (Figure 5). Subsequent abdominal computed tomography (CT), performed with and without contrast, demonstrated splenomegaly and two hypodense lesions in the right hepatic lobe, findings consistent with metastatic involvement.

Molecular analysis identified a **Q61X** mutation in the *KRAS* gene, with no detectable mutations in *NRAS* or *BRAF*. The patient received six cycles of neoadjuvant chemotherapy with 5‐fluorouracil, leucovorin, and oxaliplatin (FOLFOX regimen) in combination with bevacizumab. Follow‐up imaging demonstrated a reduction in the size of the hepatic lesions. He subsequently underwent a total colectomy with resection of the liver metastases, followed by six cycles of adjuvant chemotherapy using the same regimen.

### 2.8 Conclusion and Results (Part 3)

Pathological evaluation revealed a positive surgical margin in the hepatic lesion; therefore, close surveillance with abdominal CT imaging every 3 months was implemented for 1 year. At the time of writing, the patient remains in complete remission and continues to undergo regular clinical follow‐up.

## 3 Discussion

We present the case of a 41‐year‐old man who developed three distinct primary malignancies over a 7‐year period: acute myeloid leukemia (AML‐M2), invasive ductal carcinoma of the breast, and metastatic colorectal adenocarcinoma. His AML was initially refractory to standard induction chemotherapy but ultimately entered remission following salvage treatment with the FLAG regimen and allogeneic hematopoietic stem cell transplantation. During subsequent follow‐up, he was diagnosed with invasive ductal carcinoma of the breast, and later with adenocarcinoma of the sigmoid colon with hepatic metastases.

Despite the generally unfavorable prognosis historically associated with multiple primary malignancies, this case illustrates that such patients should not be systematically excluded from intensive or potentially curative treatment strategies. Managing a patient who sequentially develops AML, breast cancer, and colorectal cancer is inherently complex. Treatment planning must account for the cumulative effects of previous therapies, particularly the myelotoxic and systemic consequences of AML‐directed regimens. Persistent bone marrow suppression, organ toxicity, and reduced physiological reserve can significantly constrain tolerance to subsequent systemic treatments required for solid tumors. These limitations necessitate careful selection and modification of chemotherapy regimens, judicious dose adjustments, and close monitoring to mitigate severe hematologic or organ‐specific complications. Traditionally, a conservative or palliative approach has often been adopted in these complex scenarios. However, our experience challenges this paradigm. In the present case, each malignancy was managed with curative intent using tailored, evidence‐based modalities: allogeneic transplantation for AML, surgery and systemic therapy for breast cancer, and combined systemic and surgical management for metastatic colorectal cancer. Notably, the patient achieved complete remission of all three cancers.

This outcome underscores the importance of not withholding aggressive, appropriately selected therapies when they are clinically justified and compatible with the patient's overall condition. It further highlights the need for a personalized and individualized therapeutic strategy, even in the setting of multiple primary tumors.

The occurrence of multiple primary malignancies, even when metachronous, raises concern for an underlying germline susceptibility. Germline mutations in cancer‐predisposition genes—such as *BRCA1/2*, *TP53* (as in Li–Fraumeni syndrome), and DNA mismatch repair genes associated with Lynch syndrome—are well‐recognized contributors to this phenomenon [6, 7]. Although our patient did not exhibit a clear family history or classical clinical features of hereditary cancer syndromes, including Lynch syndrome, the development of three distinct primary malignancies within a relatively short time frame raises the suspicion of an underlying genetic predisposition.

This case further highlights the possibility that the development of multiple primary malignancies may be associated with an underlying genetic predisposition that extends beyond the spectrum of recognized hereditary cancer syndromes. As emphasized by Vogt et al., the presence of synchronous or metachronous primary tumors should prompt consideration of an inherited susceptibility, even in patients who do not demonstrate a distinct syndromic phenotype. In this context, comprehensive germline genetic assessment may provide clinically meaningful information by helping to clarify the biological basis of disease, support individualized treatment planning, and guide long‐term surveillance strategies. Furthermore, identifying a pathogenic germline alteration may have important implications not only for the patient but also for family members, allowing appropriate risk stratification, targeted screening, and preventive counseling in relatives at potential risk [8].

Furthermore, the history of a prior hematologic malignancy and previous exposure to intensive chemotherapeutic agents—as observed in our patient and in the cases reported by Wang et al. [9]—raises important considerations regarding both the development of subsequent primary malignancies and the complexity of managing metachronous cancers. The authors described that prolonged survival after successful treatment of hematologic malignancies, particularly acute promyelocytic leukemia, may increase the likelihood of developing secondary primary tumors, potentially related to genetic susceptibility, immune dysregulation, environmental factors, or the long‐term effects of cytotoxic therapy. In addition, prior exposure to chemotherapy may complicate subsequent oncologic management because of cumulative toxicities, impaired bone marrow reserve, and limitations in selecting further systemic treatment strategies. These risks highlight the need for robust supportive care strategies, including antimicrobial prophylaxis, growth factor support, and vigilant monitoring throughout the treatment course. The complexity of such cases underscores the importance of a multidisciplinary approach, involving hematologists, medical oncologists, surgeons, radiation oncologists, pathologists, genetic counselors, and supportive care teams. Such collaboration ensures that treatment decisions are appropriately tailored to the patient's evolving clinical status and long‐term prognosis [9, 10].

Our patient received sequential interventions, including mastectomy for breast carcinoma, neoadjuvant chemotherapy for colorectal cancer, and surgical resection of liver metastases. The decision to avoid anthracycline‐based chemotherapy for the breast malignancy, given his prior exposure to daunorubicin and the associated cardiotoxicity risk, underscores the need for individualized treatment planning in patients with a history of multiple malignancies. Similarly, the use of targeted therapy with bevacizumab for metastatic colorectal cancer reflects the growing role of molecular profiling in guiding therapeutic choices. The identification of a *KRAS* Q61X mutation in this patient further exemplifies how tumor biology can influence the selection of systemic therapies and highlights the importance of integrating molecular diagnostics into routine clinical practice.

## 4 Recommendations for Clinicians

Given the increasing number of reported cases of multiple primary malignancies, further research is needed to clarify the relationship between prior chemotherapy and the risk of subsequent malignancies. Concurrently, the development of systematic genetic screening and risk‐stratification strategies is needed to identify individuals at heightened risk of multiple malignancies. Such efforts could facilitate earlier detection, enable tailored surveillance protocols, and potentially allow for preventive interventions in high‐risk populations. Ultimately, improved understanding of the interplay between genetic susceptibility, prior therapies, and environmental factors will be essential to optimizing outcomes for patients with multiple primary cancers.

## Author Contributions

**Mohammad Hossein Anbardar:** methodology, conceptualization, supervision. **Nazanin Alemzadeh:** writing – original draft, writing – review and editing, data curation, investigation. **Abolfazl Khalafi‐Nezhad:** project administration, conceptualization, validation, methodology, supervision, resources. **Zahra Ghanbarinasab:** writing – original draft, data curation, investigation, visualization.

## Funding

The authors have nothing to report.

## Ethics Statement

The current study was approved by the Ethics Committee of Shiraz University of Medical Sciences (IR.SUMS.MED.REC.1404.332). Written informed consent was obtained from the patient for participation in this study and for publication of this case report, including all related clinical, radiologic, and pathologic images. A copy of the signed consent form is available for review by the journal editor upon request.

## Conflicts of Interest

The authors declare no conflicts of interest.

## Data Availability

The data that support the findings of this study are available on request from the corresponding author. The data are not publicly available due to privacy or ethical restrictions.
